# Editorial: Methods, techniques, and applications involving the use of high-resolution microscopy to study cell structures

**DOI:** 10.3389/fcell.2023.1323421

**Published:** 2023-11-10

**Authors:** Luis F. Jiménez-García

**Affiliations:** Department of Cell Biology, Faculty of Sciences, National Autonomous University of Mexico, México City, Mexico

**Keywords:** atomic force microscopy, confocal microscopy, electron microscopy, cell membrane, exosomes, ovary

The study of detailed cell structure is necessary for understanding function at the molecular level *in situ*. Therefore, high-resolution microscopy is useful in cell biology at the microscale and nanoscale. Cell membrane structure and its role in producing vesicles as exosomes, the organization of cell junctions and contacts to explore communication between cells, and the three-dimensional analysis of cytoplasm elements, such as mitochondria, the endoplasmic reticulum, and chromatin, both at the cellular and tissue-organ level and also at different differentiation stages, are still under investigation. This Research Topic includes papers that involve high-resolution microscopy techniques and methods applied to cell and development biology. Confocal light microscopy, dual beam electron microscopy, and atomic force microscopy are used to better understand several biological phenomena. In one paper, Sbarigia et al. used atomic force microscopy to investigate the budding process of extracellular vesicles during neurodegeneration to better understand their morphological, biophysical, and biochemical characteristics, compared with transmission electron microscopy observations. In another paper, Merchant-Larios et al. proposed a workflow chart to automate image acquisition with focused ion beam scanning electron microscopy (FIB-SEM) for large samples for more suitable serial sectioning in blocks. The use of FIB-SEM is underway as an alternative to classical serial sectioning in transmission electron microscopy. By using this technique, the authors were able to describe cell structures not previously shown in rabbit ovary cysts. They describe for the first time the fusion of oocytes in the ovary through filopodia-like processes. Similarly, Trebichalska et al. used FIB-SEM to analyze the three-dimensional structure of the cytoplasm of the human oocyte. By using this technique, they were able to visualize a more complete landscape of mitochondria, endoplasmic reticula, and cortical granules. Both papers using FIB-SEM highlight the advantages of three-dimensional reconstruction with high resolution but also point to the problem of it being a destructive technique, which can be considered a limitation. Finally, a Review describing the role of transmission electron, fluorescence, and confocal microscopy in investigating cell-to-cell interactions through the seminiferous tubule between Sertoli cells (the blood-testis barrier) during spermatogenesis has been published by Luaces et al. The authors suggest that there is a need to further use higher-resolution microscopy at super-resolution to understand the blood-testis barrier at the nanoscale. Overall, the papers highlight technologies and cutting-edge applications of high-resolution microscopy that will help improve our understanding of internal cell structures *in situ* and their role in developmental biology.

